# Assessment of Haemostasis in Disseminated Intravascular Coagulation by Use of Point-of-Care Assays and Routine Coagulation Tests, in Critically Ill Patients; A Prospective Observational Study

**DOI:** 10.1371/journal.pone.0151202

**Published:** 2016-03-09

**Authors:** Thomas Kander, Anna Larsson, Victor Taune, Ulf Schött, Nahreen Tynngård

**Affiliations:** 1 Medical Faculty, University of Lund, Lund, Sweden; 2 Department of Intensive and Perioperative Care, Skåne University Hospital Lund, 22185, Lund, Sweden; 3 Department of Clinical Immunology and Transfusion Medicine, and Department of Clinical and Experimental Medicine, Linköping University, Linköping, Sweden; 4 Department of Clinical Chemistry and Department of Clinical and Experimental Medicine, Linköping University, Linköping, Sweden; Royal College of Surgeons, IRELAND

## Abstract

**Background:**

Disseminated intravascular coagulopathy (DIC) relates to the consumption of coagulation factors and platelets with bleeding and micro thrombosis events.

**Aim:**

The aim of this study was to compare haemostasis parameters in critically ill patients with DIC versus patients without DIC, and in survivors versus non-survivors over time. Correlations between the DIC-score, the degree of organ failure and the haemostasis were assessed.

**Method:**

Patients admitted to the intensive care unit with a condition known to be associated with DIC and with an expected length of stay of >3 days were included. Routine laboratory tests, prothrombin time, activated partial thromboplastin time, platelet count, fibrinogen concentration and D-dimer were measured. Coagulation and platelet function were assessed with two point-of-care devices; Multiplate and ROTEM. DIC scores were calculated according to the International Society on Thrombosis and Haemostasis and Japanese Association for Acute Medicine.

**Results:**

Blood was sampled on days 0–1, 2–3 and 4–10 from 136 patients with mixed diagnoses during 290 sampling events. The point-of-care assays indicated a hypocoagulative response (decreased platelet aggregation and reduced clot strength) in patients with DIC and, over time, in non-survivors compared to survivors. Patients with DIC as well as non-survivors had decreased fibrinolysis as shown by ROTEM. DIC scores were higher in non-survivors than in survivors.

**Conclusions:**

Patients with DIC displayed signs of a hypocoagulative response and impaired fibrinolysis, which was also evident over time in non-survivors. Patients with DIC had a higher mortality rate than non-DIC patients, and DIC scores were higher in non-survivors than in survivors.

## Introduction

Coagulopathy is common in patients in the intensive care unit (ICU). There are multiple contributing factors, such as impaired synthesis or increased consumption of coagulation factors and platelets due to massive bleeding, for example, as well as platelet dysfunction [1] and derangement of fibrinolysis. This can lead to a serious condition with microvascular thrombosis: disseminated intravascular coagulopathy (DIC) and can result in multiple organ failure and mortality.

Two scoring algorithms are frequently used for the diagnosis of DIC: the International Society on Thrombosis and Haemostasis (ISTH) score and the Japanese Association for Acute Medicine (JAAM) score [2]. DIC is associated with a prolongation of activated partial thromboplastin time (aPTT) and prothrombin time (PT) [3–5], which are two methods routinely used to monitor coagulation. However, neither of these tests nor the measurement of fibrinogen or platelet count are adequate to detect coagulopathy and DIC [6]. Newer, point-of-care (POC) methods have been introduced in the intensive care and perioperative settings. These include the viscoelastic haemostatic assays (VHAs) such as rotational thromboelastometry (ROTEM; Pentapharm, Munich, Germany) and multiple electrode aggregometry (Multiplate; Roche Diagnostics GmbH, Mannheim, Germany). VHA monitors coagulation in whole blood by determining clotting time and clot structure (clot strength), variables affected by coagulation factors and platelets [7]. Multiplate measures the platelets ability to respond to stimulation with different agonists by aggregation measurement [7]. VHA is currently regarded as the best option when monitoring coagulation in perioperative patients [8] and can be used to monitor bleeding and to guide transfusion therapy [9–11]. Both ROTEM and Multiplate have been shown to predict mortality in trauma patients [12–14]. We recently demonstrated a hypocoagulative response measured with POC assays including ReoRox (Medirox AB, Nyköping, Sweden), another VHA instrument, in non-surviving, critically ill patients compared to survivors [15]. However DIC incidence and its relationship to POC results were not investigated in that study. To our knowledge only a few studies have investigated coagulation and platelet function using ROTEM and Multiplate in patients with DIC [16–18] and only one of them used both instruments [16].

The aim of this prospective observational study was to compare haemostasis in critically ill patients with DIC versus patients without DIC using Multiplate, ROTEM and routine coagulation tests and to investigate correlations between DIC scores, the degree of organ failure and haemostasis. An additional aim was to assess haemostasis in survivors compared to non-survivors over time.

## Materials and Methods

### Study subjects and blood sampling

Patients admitted to the ICU at Skåne University Hospital in Lund with a condition known to be associated with DIC and with an expected length of stay >3-days were included in the study. The study was approved by the Regional Ethical Review Board (Lund, Protocol DNR 2010/482 and DNR 2014/916). Informed and signed consent was obtained from all patients or their next of kin.

Blood samples were obtained within 10 days after admission to the ICU (median time to the first sampling occasion was 1.0 days). Of the patients, 65% had the first sample taken at day 0–1, 24% at days 2–3, and the remainder at days 4–10. In total, 18% of the patients had a follow-up sample taken at days 2–3 and 19% at days 4–10. Blood was drawn through an arterial catheter using a Safedraw PMSET 1DT, (Argon Critical Care Systems, Singapore) and collected in Vacutainer tubes (Becton Dickinson, Plymouth, UK) with citrate (0.129 M) as the anticoagulant for thromboelastography and in a 3.0 mL Hirudin tube (Roche Diagnostics) for aggregometry with Multiplate.

### Routine laboratory assays

Routine laboratory assays, PT (Owren method, not Quick) with international normalized ratio (PT-INR), aPTT, platelet count, fibrinogen concentration and D-dimer were determined according to the accredited methods at the Department of Clinical Chemistry at the University Hospital in Lund.

### Multiple electrode aggregometry

Platelet aggregation was assessed by impedance technology using Multiplate. Multiplate measures platelet adhesion to the electrodes in the test cuvette after stimulation of the platelets with a platelet agonist. The adhesion of the platelets to the electrodes changes the electric resistance between the electrodes, which is detected. In the analysis, 300 μL of pre-warmed 9 mg/mL NaCl (B. Braun, Melsungen, Germany) was added to the test cuvette, followed by 300 μL of hirudin anticoagulated blood. The blood and buffer were incubated under constant stirring for 3 min. This was followed by the addition of 20 μL of platelet agonists adenosine-5´-diphosphate (ADP), collagen and thrombin receptor agonist peptide (TRAP), respectively (ADPtest, COLtest, or TRAPtest; Roche Diagnostics) and the aggregation response followed for 6 min at 37°C under constant stirring. The final concentrations of the agonists were 32 μM for the TRAPtest, 6.5 μM for the ADPtest and 3.2 μg/mL for the COLtest. The area under the curve (AUC) was determined and used as a measure of aggregation.

Aggregometry with Multiplate was assessed on blood obtained from 131 of the patients (284 sampling occasions). The TRAPtest was not included as an agonist until later in the study; therefore, only 122 patients were assessed with TRAP (276 sampling occasions). All samples were analysed in the ICU laboratory within 0.5–3.0 hours from blood collection as recommended by the manufacturer, which resulted in a median time to analysis of 40 min.

### Viscoelastic coagulation analysis

Thromboelastography (ROTEM) was used to measure clot formation and clot elasticity. ROTEM has a fixed sample cup with a pin suspended in the blood sample. The pin oscillates, and the movement is registered in the coagulating sample [19], which gives rise to a curve. Several variables are obtained from the curve, including the clot time (CT), the maximum clot firmness (MCF; the maximum strength/stiffness of the clot), and the maximum clot lysis (ML) [19]. After the addition of 20 μL of 0.2M CaCl_2_ (StartTEM) to 300 μL of blood, coagulation was initiated by thromboplastin alone (ExTEM) in the presence of cytochalasin D (FibTEM). Cytochalasin D inhibits platelet function; therefore, FibTEM provides information on the functional fibrinogen concentration and fibrin stability of the clot. CT and MCF variables were determined from the ExTEM tracings and MCF variables were determined from the FibTEM tracings. ML was determined in 242 ExTEM samples. All samples were analysed within 3.5 hours of blood collection (median 10 min) at 37°C in the ICU laboratory.

### ICU scores

There are different scoring systems commonly used to assess the prognosis and severity of the disease and to predict a patient's outcome in the ICU.

The Simplified Acute Physiology Score 3 (SAPS3) score provides an estimate on the degree of affecting diseases upon admission to the ICU [20]. The expected mortality rate (EMR) is calculated from the SAPS3. The sequential organ failure assessment (SOFA) score was developed to better describe the degree of organ failure with a simple scoring system [21] and is calculated daily. Based on six organ systems (cardiovascular, respiratory, hepatic, renal, coagulation and neurological), the SOFA is used to estimate the degree of organ dysfunction. Every organ system is given a point from 1 to 4, where a higher score indicates a greater degree of organ failure. Platelet count represents the status of the coagulation system. Patient mortality statistics 30 days after admission to the ICU, SOFA scores and SAPS3 scores were retrieved from the regular patient administrative system (PASIVA). DIC scores, according to ISTH and JAAM, were used to define the presence of DIC. ISTH and JAAM DIC scores were calculated as described by Takemitsu et al. [22]. To calculate JAAM, the systemic inflammatory response syndrome (SIRS) criteria were determined for all patients and sampling occasions. Five points or more indicated overt DIC according to the ISTH definition, and four points or more indicated DIC according to the JAAM definition. SOFA, ISTH and JAAM DIC scores could not be calculated for six patients (seven sampling occasions) due to missing laboratory data.

### Statistical analyses

Statistical analyses were performed using SPSS (IBM SPSS Statistics version 22, IBM Corporation, Armonk, NY, USA). The variables did not meet the criteria for normal distribution by the Shapiro-Wilks test of normality. All variables were summarized using the median with the 25th to 75th percentiles as the distribution measurement shown in parentheses. The Mann-Whitney U test was used to calculate the differences between patients with or without DIC, as determined by the JAAM score, and between survivors and non-survivors. Data were considered significant when the p-value was <0.05. Correlation coefficients (r_s_) were calculated using Spearman's rank correlation for non-parametric tests.

## Results

### Patient demographics

Patient demographics are presented in Table 1. In all, 136 patients (84 males and 52 females with a median age of 65 years) with POC and routine coagulation tests up to 10 days after ICU admission were included in the study; 290 sampling events from these patients were used for correlations and subgroup analyses.

**Table 1:** Patient demographics.

	All	Survivors	Non-survivors
	(n = 136)	(n = 98)	(n = 38)
**Patients in the diagnosis groups** (n)			
*Sepsis*	33	22	11
*Trauma*	20	19	1
*Medical*	43	30	13
*Surgery*	29	24	5
*Cardiac arrest*	11	3	8
**30 day mortality** (%)	30		
**EMR** (%)	40		
**Days at intensive care unit**	4 (2–7)	4 (2–7)	3 (2–5)
**SOFA score**	7 (5–11)	7 (4–10)**	10 (6–16)
**SAPS score**	61 (51–73)	57 (48–66)**	74 (65–82)
**ISTH DIC score**	2 (1–3)	2 (1–2)**	2 (1–4)
**JAAM DIC score**	2 (1–4)	2 (1–3)**	3 (2–5)
**Overt DIC** (ISTH-score; %)		3**	18
**DIC** (JAAM-score; %)		22**	47
**Anticoagulation** (%)	47	49	42
**Platelet inhibitors** (%)	14	9**	28
**Patients transfused** (%)			
*Plasma*	16	16	16
*Red blood cells*	49	49	50
*Platelets*	13	13	16

The number of patients included in the study categorised into 5 diagnosis groups, estimated mortality risk (EMR), the number and percentage of patients with actual 30-day mortality, the percentage of patients with overt disseminated intravascular coagulopathy (DIC) determined by ISTH-score and JAAM-score as well as percentage of patients on anticoagulants and platelet inhibitors and transfused within 24h of blood collection. Results for days in the intensive care unit, sequential organ failure assessment (SOFA), Simplified Acute Physiology Score 3 (SAPS3), ISTH DIC score and JAAM DIC score are presented as medians with the 25^th^–75^th^ percentiles shown within parentheses. All data from the sampling occasions were included (n = 290 for all patients, n = 214 for survivors, n = 76 for non-survivors except SOFA, ISTH DIC and JAAM DIC score where n = 283 for all patients, n = 76–74 for non-survivors and n = 209–207 for survivors, and SAPS3 which was only assessed once for each patient).

** = p <0.01 compared to non-survivors.

The overall 30-day mortality was 28%, whereas EMR predicted 40% mortality. SOFA and SAPS3 were increased in non-survivors. On at least one of the sampling occasions, 8% of the patients had overt DIC by ISTH score definition (≥5 point score) and 29% had DIC as assessed by the JAAM score definition (≥4 point score). A larger proportion of non-survivors had DIC as assessed by both scoring algorithms (Table 1). The use of platelet inhibiting pharmaceuticals and platelet transfusions was generally low (Table 1), but patients with DIC had a higher incidence of transfusions with plasma, platelets and red blood cells (p <0.05; data not shown).

### Comparisons of patients with DIC compared to non-DIC patients

When dividing patients into DIC or non-DIC groups, the JAAM DIC definition was used. Patients with DIC had significantly higher SOFA and SAPS3 scores and a longer stay in the ICU than patients without DIC (Table 2).

**Table 2:** Patient characteristics with and without DIC.

	JAAM DIC	JAAM Non-DIC
	(n = 38)	(n = 92)
**Days at intensive care unit**	5 (2–10)	3 (2–5
**30 day mortality** (%)	45	23*
**SOFA score**	12 (8–16)	6 (4–8)**
**SAPS score**	68 (59–82)	60 (46–67)**
**ISTH DIC score**	3 (2–4)	1 (1–2)**
**JAAM DIC score**	5 (4–6)	2 (1–2)**

Results for patients with and without disseminated intravascular coagulopathy (DIC) determined by JAAM score algorithm. Mortality is presented as percentage. Results for days in the intensive care unit, sequential organ failure assessment (SOFA), Simplified Acute Physiology Score 3 (SAPS3), ISTH DIC score and JAAM DIC score are presented as medians with the 25^th^–75^th^ percentiles shown within parentheses. Data from all sampling occasions were included (n = 101 for DIC patients and n = 182 for non-DIC patients except for SOFA where n = 177 for non-DIC patients and SAPS3 which was only assessed once for each patient).

* = p <0.05 and

** = p <0.01 compared to patients with DIC.

PT-INR and D-dimer were higher for patients with DIC and fibrinogen and platelet count lower. aPTT was also longer in patients with DIC. The median ADP-, COL-, and TRAP-induced aggregation response for patients with DIC were below the reference range and was significantly lower than for patients without DIC. ROTEM analysis showed that patients with DIC had significantly longer clot time (CT), lower elasticity (MCF for both ExTEM and FibTEM) as well as lower ML compared to non-DIC patients. However, these values were still within the normal range (Table 3).

**Table 3:** Routine laboratory assay variables and POC assay variables in patients with or without DIC.

	All patients	JAAM DIC	JAAM Non-DIC	Reference values
	(n = 136)	(n = 38)	(n = 92)	
**Routine laboratory variables**				
Platelet count (x 10^9^ /L)	154 (102–230)	76 (31–130)	188 (140–264)**	Male 145–348 Female 165–387
aPTT (sec)	33 (30–40)	38 (32–45)	32 (29–38)**	26–33
PT (INR)	1.3 (1.2–1.4)	1.4 (1.3–1.6)	1.3 (1.1–1.4) **	0.9–1.2
Fibrinogen (g/L)	4.5 (3.4–6.1)	4.3 (3.2–5.7)	4.7 (3.7–6.4)**	2.0–4.0
D-dimer (mg/L)	1.2 (0.6–2.8)	2.5 (1.1–4.2)	0.9 (0.5–2.1)**	<0.25
**Multiplate variables**				
ADP-AUC (U)	58 (32–88)	34 (9–74)	67 (40–93)**	57–113
COL-AUC (U)	79 (57–107)	61 (19–105)	84 (69–111)**	72–125
TRAP-AUC (U)	97 (67–127)	74 (26–121)	104 (77–127)**	84–128
**ROTEM variables**				
CT (sec)	64 (55–80)	71 (57–99)	63 (54–73)**	38–79
MCF-ExTEM (mm)	65 (58–70)	56 (40–64)	67 (63–72)**	50–72
ML-ExTEM (%)	8 (4–12)	5 (0–8)	9 (6–13)**	<15
MCF-FibTEM (mm)	23 (16–32)	19 (12–28)	26 (19–35)**	9–25

Routine laboratory variables platelet count, activated partial thromboplastin time (aPTT), prothrombin time (PT), fibrinogen and d-dimer and point-of-care (POC) variables for Multiplate and ROTEM for all patients and patients with or without disseminated intravascular coagulopathy (DIC) according to the JAAM scoring algorithms (DIC defined as a score of ≥4 on at least 1 sampling occasion). Results for Multiplate are presented as area under the curve (AUC) after stimulation with adenosine-5´-diphosphate (ADP), collagen (COL) and thrombin receptor agonist peptide (TRAP) and for ROTEM as clot time (CT), maximum clot firmness (MCF) and maximum clot lysis (ML). Results are presented as medians with the 25^th^–75^th^ percentiles shown within parentheses. Results for all sampling occasions were included (n = 290 for all patients, n = 101 for DIC patients and n = 182 for non-DIC patients except for aPTT, fibrinogen, D-dimer, ADP, COL, TRAP where n = 284–276 for all patients, n = 101–95 for DIC patients and n = 180–175 for non-DIC patients. For ML n = 242 for all patients, n = 77 for DIC patients and n = 161 for non-DIC patients).

** = p <0.01 compared to patients with DIC.

### Comparisons between survivors and non-survivors

Non-survivors had significantly prolonged aPTT from days 2–3 as well as reduced platelet count and fibrinogen concentrations on days 4–10. The aggregation response in non-survivors was lower than for survivors on days 4–10, with values below the normal range. CT, as detected with ROTEM, was prolonged in non-survivors compared to survivors early in the ICU stay but was similar at later stages. In contrast, the clot elasticity (MCF) and fibrinolysis variables (ML) were the same between non-survivors and survivors at the beginning of the ICU stay, but on days 4–10 the clot elasticity and fibrinolysis in non-survivors was lower compared to survivors but the elasticity still remained within the normal range in non-survivors (Table 4).

**Table 4:** Routine laboratory assay variables and POC assay variables in survivors and non-survivors.

	Day 0–1		Day 2–3		Day 4–10	
	Survivors	Non-survivors	Survivors	Non-survivors	Survivors	Non-survivors
	(n = 61–56^a^)	(n = 25–22^b^)	(n = 39–35^c^)	(n = 18^d^)	(n = 31–28)	(n = 10^e^)
**Routine laboratory variables**						
Platelet count (x 10^9^ /L)	146 (114–200)	150 (84–244)	145 (108–191)	110 (50–209)	207 (146–266)**	23 (15–130)
aPTT (sec)	36 (30–45)	38 (31–46)	34 (30–38)*	39 (36–47)	30 (29–34)	41 (36–45)
PT (INR)	1.3 (1.1–1.4)	1.3 (1.1–1.5)	1.3 (1.2–1.5)	1.4 (1.3–1.5)	1.3 (1.1–1.4)	1.4 (1.3–1.5)
Fibrinogen (g/L)	3.5 (2.7–4.8)	3.8 (2.2–6.2)	4.7 (4.0–6.1)	4.6 (3.7–5.4)	5.3 (4.4–7.2)	3.9 (3.3–4.3)
D-dimer (mg/L)	1.2 (0.6–2.5)	2.0 (0.7–4.6)	1.4 (0.6–2.1)	1.2 (0.4–2.4)	1.7 (0.7–2.9)	0.8 (0.6–4.6)
**Multiplate variables**						
ADP-AUC (U)	52 (34–79)	75 (61–94)	47 (33–65)	38 (26–96)	66 (48–92)*	19 (7–60)
COL-AUC (U)	75 (61–98)	81 (63–101)	75 (55–92)	75 (52–98)	92 (73–113)*	35 (6–84)
TRAP-AUC (U)	95 (72–117)	120 (86–135)	81 (69–121)	78 (65–142)	116 (76–129)**	28 (13–89)
**ROTEM variables**						
CT (sec)	61 (50–74)*	80 (58–95)	66 (50–75)	67 (60–101)	65 (59–80)	74 (62–93)
MCF-ExTEM (mm)	61 (57–66)	63 (56–69)	64 (59–68)	63 (57–69)	68 (65–74)**	43 (35–62)
ML-ExTEM (%)	9 (5–12)	6 (3–11)	9 (5–15)	8 (5–11)	7 (6–11)**	0 (0–3)
MCF-FibTEM (mm)	28 (13–27)	20 (13–25)	22 (18–30)	24 (17–27)	30 (26–40)**	20 (15–27)

Routine laboratory variables platelet count, activated partial thromboplastin time (aPTT), prothrombin time (PT), fibrinogen and d-dimer and point-of-care (POC) variables for Multiplate and ROTEM in survivors and non-survivors. Results for Multiplate are presented as area under the curve (AUC) after stimulation with adenosine-5´-diphosphate (ADP), collagen (COL) and thrombin receptor agonist peptide (TRAP) and for ROTEM as clot time (CT), maximum clot firmness (MCF) and maximum clot lysis (ML). Results are presented as medians for day 0–1, day 2–3 and day 4–10. The 25^th^–75^th^ percentiles are shown inside parentheses.

* = p <0.05 and

** = p <0.01 compared to non-survivors.

^a^ n = 48

^b^ n = 18

^c^ n = 32

^d^ n = 15 and

^e^ n = 6 for ML-ExTEM.

### Correlation between assays

**Fig 1:**
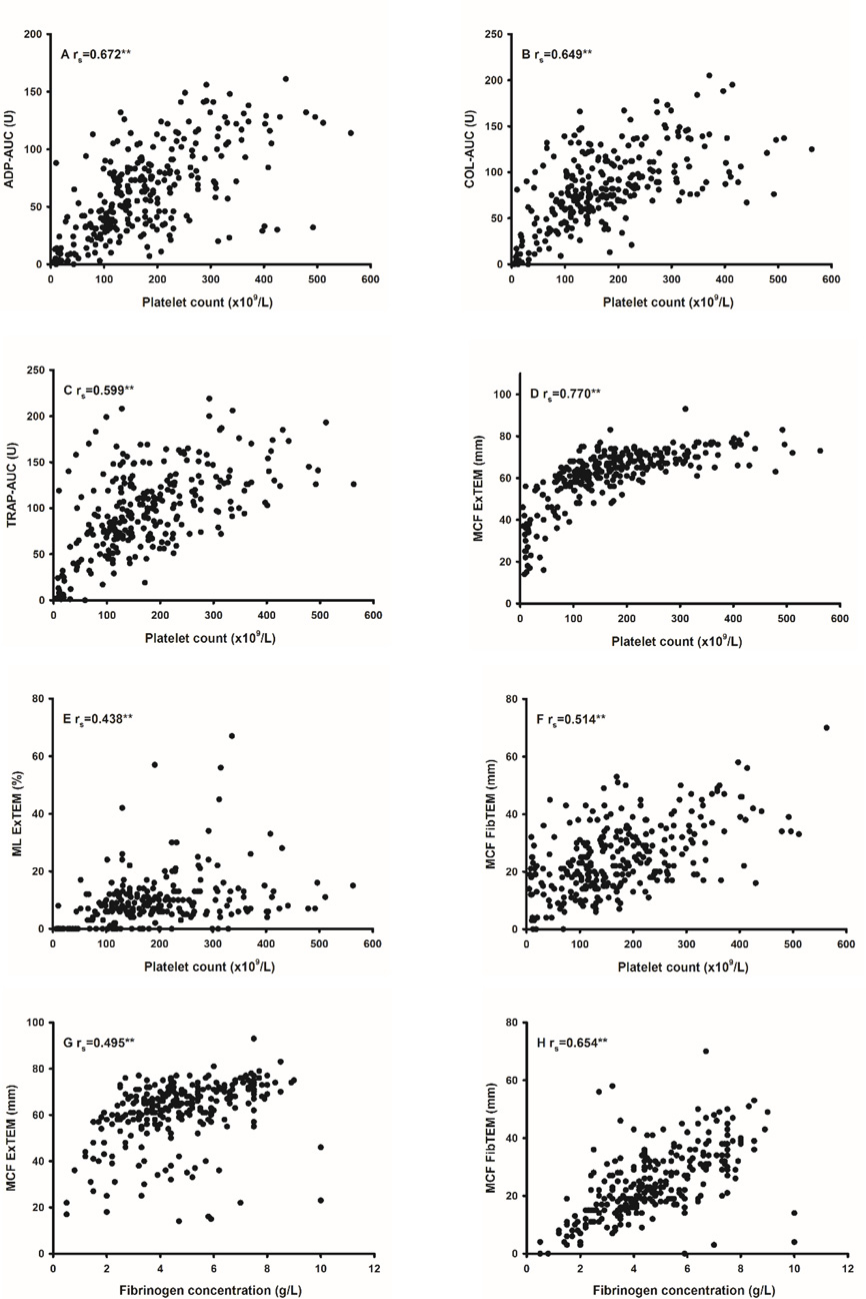
Correlations between routine laboratory assays and POC assays. Correlations (r_s_) between platelet count and Multiplate area under the curve (AUC) for adenosine-5´-diphosphate (ADP) (A), collagen (B), thrombin receptor agonist peptide (TRAP) (C) and ROTEM maximum clot firmness (MCF) for ExTEM (D), maximum clot lysis (ML) for ExTEM (E) and MCF for FibTEM (F). Correlations also shown between fibrinogen concentration and MCF for ExTEM (G) and MCF for FibTEM (H). ** = p <0.01.

Only weak or no correlations could be detected between POC assay variables and routine assay variables PT-INR, aPTT and D-dimer (r_s_ <0.4). The Multiplate aggregation response, after stimulation with all agonists, correlated significantly to PLC (Fig 1), but not to fibrinogen.

The ROTEM clot elasticity (MCF for ExTEM) correlated with the PLC (p <0.01) and increased when the PLC increased (Fig 1). Increasing concentrations in fibrinogen also increased the fibrinogen component of clot elasticity (MCF for FibTEM) (Fig 1; p <0.01). The Multiplate variables showed poor correlation (r_s_ <0.4) to both the SOFA score and the DIC scores (Table 5).

**Table 5:** Correlation between ICU-scores (SOFA and DIC-scores) and POC assays.

	SOFA	ISTH DIC	JAAM DIC
**ADP-AUC**	-0.360**	-0.360**	-0.365**
**COL-AUC**	-0.349**	-0.280**	-0.303**
**TRAP-AUC**	-0.329**	-0.279**	-0.346**
**CT-ExTEM**	0.330**	0.279**	-0.346**
**MCF-ExTEM**	-0.420**	-0.515**	-0.561**
**ML-ExTEM**	-0.328**	-0.358**	-0.386**
**MCF-FibTEM**	-0.200**	-0.329**	-0.223**

Correlations (r_s_) between intensive care unit (ICU) scores and point-of-care (POC) assay variables. ICU-scores presented are sequential organ failure assessment (SOFA and disseminated intravascular coagulopathy (DIC) scores according to the JAAM and ISTH scoring algorithms. POC assay variables are area under the curve (AUC) after stimulation with adenosine-5´-diphosphate (ADP), collagen (COL) and thrombin receptor agonist peptide (TRAP), respectively, and clot time (CT), maximum clot firmness (MCF) for ExTEM and FibTEM, respectively, and maximum clot lysis (ML).

** = p <0.01.

**Fig 2:**
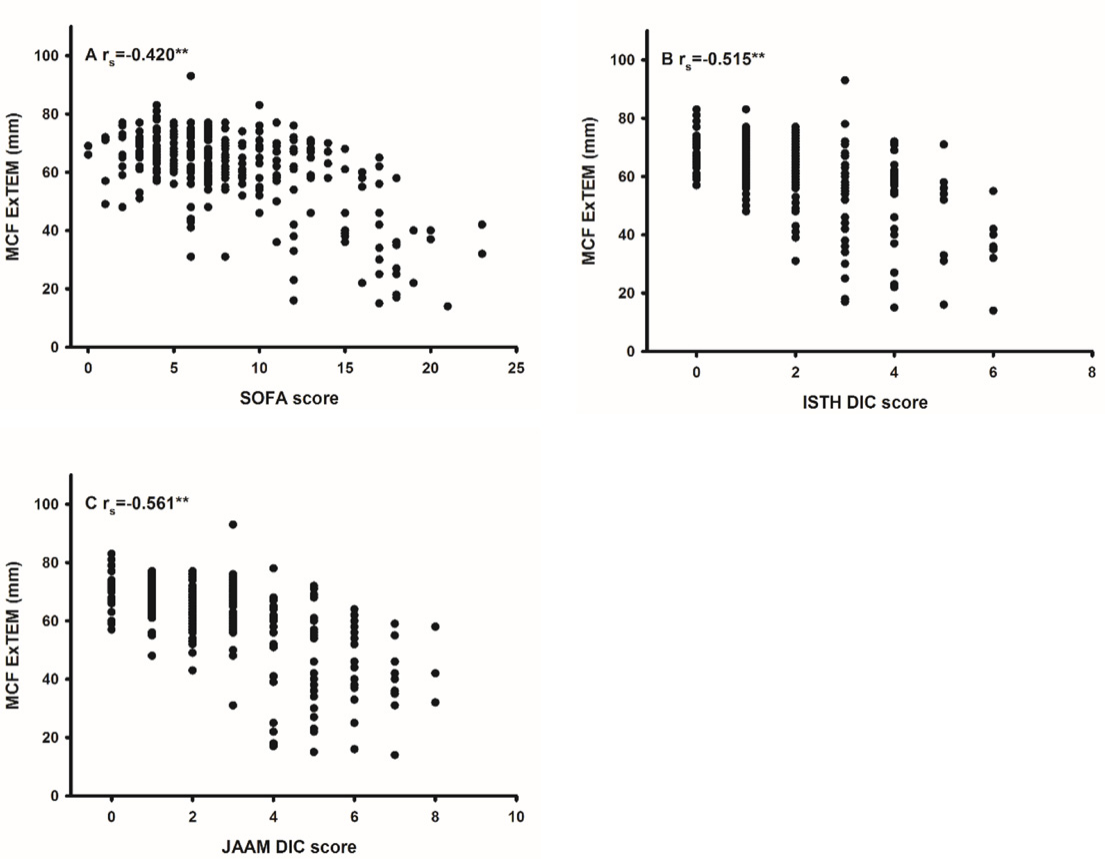
Correlations between ROTEM and ICU-scores. Correlations (r_s_) between ROTEM maximum clot firmness (MCF) for ExTEM and intensive care unit (ICU) scores. Sequential organ failure assessment (SOFA) score (A), disseminated intravascular coagulopathy (DIC) scores according to ISTH (B) and JAAM (C). ** = p <0.01.

Of the POC variables, MCF for EXTEM showed the best correlation (Fig 2, Table 5).

## Discussion

In this prospective observational study in critically ill patients with mixed diagnosis, we found that a higher proportion of patients had DIC when assessed using the JAAM score compared to the ISTH score. Mortality was higher in patients with DIC than in patients without DIC. Patients with DIC, as well as non-survivors, showed a hypocoagulative response when assessed by the POC devices Multiplate and ROTEM. Fibrinolysis, measured with ROTEM, was found to be impaired in non-survivors and in patients with DIC.

POC coagulation devices have gained widespread use in the perioperative setting. This study investigated the differences in haemostasis in critically ill patients using two POC coagulation devices and routine laboratory assays. This category of patients was further divided into those with DIC and those without DIC, and into survivors and non-survivors.

Individually, the routine laboratory variables PT, platelet count, fibrinogen and D-dimer are less useful in detecting DIC; therefore, these variables were combined in the ISTH and JAAM scoring algorithms used to detect DIC [23]. In the present study, more patients had DIC defined by the JAAM algorithm compared to the ISTH algorithm. This could be due to a higher sensitivity of JAAM score than the ISTH score, as suggested previously [2, 24] or that it is less stringent in assigning points toward DIC diagnosis. The JAAM score, in addition to the above routine variables, uses the SIRS criteria to determine the score [2]. Furthermore, the JAAM score excludes the fibrinogen concentration in the scoring algorithm [2]. It is possible that these differences might have contributed to the higher proportion of patients with DIC according to the JAAM score compared to the ISTH-score. A high DIC score has been shown to be associated with a high incidence of massive bleeding and mortality [2, 25, 26], and, as shown in our study, to be associated with a longer stay in the ICU [25]. We also found higher mortality of patients with DIC and higher DIC scores in non-survivors. It has recently been suggested that the use of the fixed ISTH score of ≥5 misses many patients with life-threatening DIC [25]. To assign patients to DIC and overt DIC a score of ≥ 4 for JAAM and ≥5 for ISTH, respectively, at any time point during the 10 day period was used in our study and a similar strategy was used in a recent study [25]. This could have led to an overestimation of DIC and overt DIC, respectively.

ROTEM allows the coagulation to be monitored in whole blood, thus coagulation is assessed in the presence of all blood cells, as in the circulation. PT and aPTT in contrast uses only plasma for coagulation measurement. In addition ROTEM allows fibrinolysis to be determined. We found the clotting time as measured by ROTEM to be prolonged in patients with DIC, thus corresponding to the increased PT in DIC patients. Previous studies on patients with sepsis reported that patients with DIC showed signs of hypocoagulation, including low aggregation response and clot elasticity [16, 17, 27]. Also, in this study, DIC was found to be associated with decreased clot elasticity (both MCF for ExTEM and FibTEM) as detected by ROTEM. The lower clot elasticity in patients with DIC was most likely in part accounted for by the lower PLC, as we and others found that the clot elasticity correlated with PLC [28]. The lower fibrinogen concentration in patients with DIC compared to non-DIC patients could also in part be responsible for the lower clot elasticity as this marker also correlated to clot elasticity. Interestingly, the fibrinogen concentration was approximately 10% lower in patients with DIC, however, this decrease resulted in a nearly 30% lower MCF in FibTEM assay. In contrast patients without DIC showed a tendency of higher MCF FiBTEM (median of 26 and 25–75^th^ percentile of 19–35) than reference range (9–25). This may be caused by the high fibrinogen value found in those patients. A high fibrinogen concentration will result in a high clot firmness in both the FibTEM and ExTEM assay even though the FibTEM assay is more affected by the fibrinogen concentration since the ExTEM is also affected by the platelet count. A decrease in ROTEM ML is reported to be a predictor of overt DIC [18], which was also the case in our study. Furthermore, we found fibrinolysis to be impaired in non-survivors compared to survivors. Decreased fibrinolysis is related to elevated levels of PAI-1 and might contribute to microcirculatory fibrin deposition and organ failure [29]. Hypo-fibrinolysis can be difficult to quantify. The reference range for ROTEM has only been established to detect hyper-fibrinolysis since it only states that ML should be <15%. A recent study by Panigada et al. with TEG (another VHA instrument) used a modified assay with urokinase to detect hypo-fibrinolysis. Urokinase initiated fibrinolysis in blood from healthy volunteers but in blood from sepsis patients, fibrinolysis was markedly impaired. The same phenomenon could not be as clearly detected using the traditional TEG assay [30].

Of the POC assay variables, ROTEM^®^ MCF with ExTEM as an agonist showed the best correlations (r_s_ of –0.515 to –0.561) to the two DIC scores. Multiplate, in contrast, showed only poor correlations. Despite the poor correlations with Multiplate, the aggregation response to all tested agonists was found to be lower in DIC patients than in non-DIC patients. Furthermore, of the two POC assays, only Multiplate showed median values below the reference range in the DIC patients. Also, here the lower PLC in DIC patients could account for this difference, since in line with previous studies [28, 31] significant correlations between the aggregation response and low PLC were found. Brenner et al. also reported significantly lower aggregation response in patients with DIC compared to non-DIC patients and compared to healthy volunteers [16]. The POC devices, in comparison to routine laboratory coagulation assays, analyse coagulation in whole blood as opposed to plasma, but none of the POC tests evaluated in this study assessed coagulation under flow conditions corresponding to the shear rates of 330–1100/s found in the venous and arterial circulation [32], nor in the presence of endothelial cells [33].

A prolonged stay at the ICU was associated with an increase in aPTT and a decrease in both PLC and fibrinogen concentration in non-survivors. Decreased fibrinogen and PLC in ICU patients have previously been reported to be associated with mortality [12, 34, 35]. A prolonged stay in the ICU also resulted in a lower aggregation response in non-survivors compared to survivors. Similar findings in patients with trauma have also been reported [12, 14]. This could partly be due to a higher use of anti-platelet therapy in non-survivors compared to survivors, but most likely it is also caused by the lower PLC in non-survivors. ROTEM revealed a prolongation in CT and a lower clot elasticity in non-survivors compared to survivors. The change in CT did not correspond to a similar change in PT, suggesting that these assays are not always comparable. CT was altered only at the beginning of the ICU stay; in contrast, the clot elasticity only decreased at later stages of the ICU stay. Low elasticity has also previously been associated with increased mortality when assessed by VHAs [6, 13, 36, 37]. Despite these differences between survivors and non-survivors, both the CT and the clot elasticity variables remained within the normal range in non-survivors.

### Limitations

Due to incomplete sampling for routine laboratory tests, SOFA scores and DIC scores could not be calculated for all patients. Our intent was to include patients as early as possible after admission to the ICU and then analyse coagulation for up to 10 days after admission to the ICU. For logistic reasons it was difficult to obtain and analyse samples on the day of admission and repeated samplings were not always possible. Furthermore, only a few patients had a follow-up sample analysed on days 4–10 after admission to the ICU, which in part can be accounted for by the short stay most patients had at the ICU (median of 4 days) due to death, or discharge. This limited the data available for longitudinal analysis.

## Conclusions

Both POC assays, ROTEM and Multiplate, showed a hypocoagulative response in patients with DIC and, over time, in non-survivors compared to survivors. DIC scores were higher in non-survivors, and patients with DIC had higher mortality.

## Supporting Information

S1 Data Data supporting the conclusion. (XLS)
